# Conditions and barriers for quality improvement work: a qualitative study of how professionals and health centre managers experience audit and feedback practices in Swedish primary care

**DOI:** 10.1186/s12875-021-01462-4

**Published:** 2021-06-14

**Authors:** Eva Arvidsson, Sofia Dahlin, Anders Anell

**Affiliations:** 1 Futurum, Region Jönköping County, Sweden; School of Health and Welfare, Jönköping University, Jönköping, Sweden; 2 Futurum, Region Jönköping County, Jönköping, Sweden; 3 Lund University School of Economics & Management, Lund, Sweden

**Keywords:** Primary health care, General practice, Quality improvement, Audit & feedback, Quality indicators, Reimbursement, Incentive

## Abstract

**Background:**

High quality primary care is expected to be the basis of many health care systems. Expectations on primary care are rising as societies age and the burden of chronic disease grows. To stimulate adherence to guidelines and quality improvement, audit and feedback to professionals is often used, but the effects vary. Even with carefully designed audit and feedback practices, barriers related to contextual conditions may prevent quality improvement efforts. The purpose of this study was to explore how professionals and health centre managers in Swedish primary care experience existing forms of audit and feedback, and conditions and barriers for quality improvement, and to explore views on the future use of clinical performance data for quality improvement.

**Methods:**

We used an explorative qualitative design. Focus groups were conducted with health centre managers, physicians and other health professionals at seven health centres. The interviews were audio recorded, transcribed and analysed using qualitative content analysis.

**Results:**

Four different types of audit and feedback that regularly occurred at the health centres were identified. The main part of the audit and feedback was “external”, from the regional purchasers and funders, and from the owners of the health centres. This audit and feedback focused on non-clinical measures such as revenues, utilisation of resources, and the volume of production. The participants in our study did not perceive that existing audit and feedback practices contributed to improved quality in general. This, along with lack of time for quality improvement, lack of autonomy and lack of quality improvement initiatives at the system (macro) level, were considered barriers to quality improvement at the health centres.

**Conclusions:**

Professionals and health centre managers did not experience audit and feedback practices and existing conditions in Swedish primary care as supportive of quality improvement work. From a professional perspective, audit and feedback with a focus on clinical measures, as well as autonomy for professionals, are necessary to create motivation and space for quality improvement work. Such initiatives also need to be supported by quality improvement efforts at the system (macro) level, which favour transformation to a primary care based system.

## Background

Good quality primary care (PC) has been associated with a number of positive outcomes such as lower rates of avoidable mortality, improved equity in health, and lower health care costs [1–3]. Against this background, most countries are trying to base their health care systems on PC. Contemporary PC systems across OECD countries are expected to take the main responsibility for first-line care, chronic care, and coordination of care performed by others. Expectations on PC are rising as societies age and the burden of chronic disease grows [4]. In many countries, PC is trying to adapt by transforming into multidisciplinary team work, in particular to improve care for patients with chronic diseases and multimorbidity [5, 6].

Despite the ambition to strengthen the role of PC, its share of health care resources is declining in the OECD [7, 8]. The proportion of general practitioners (GPs) in relation to the total number of physicians across OECD countries dropped from 32 to 29 per percent between 2000 and 2016 [8]. The WHO 2008 report “Primary health care: now more than ever”, concluded that “left to their own devices, health systems do not gravitate naturally towards the goals of health for all through primary health care” and “Health systems are developing in directions that contribute little to equity and social justice and fail to get the best health outcomes for their money.”

To increase equity in health and access to care, a new PC model with publicly owned health centres employing a multidisciplinary workforce was introduced in Sweden in the late 1960s. Since then, Swedish PC has been the target of several reforms and changes, but the focus on a multidisciplinary workforce has remained unchanged. Increased patient choice and privatisation of providers was initiated by regional and national governments in 2007–2010 [9, 10]. The idea was that if (owners of) HCs competed with each other for health professionals and patients, the competition would make them perform better, which in turn would increase the quality and efficiency of PC [11]. The outcome of these reforms is debated and the results from studies are inconclusive [12, 13]. Overall, however, studies indicate that reforms have had little impact on the capacity and quality of PC [14]. A low proportion of Swedish physicians are GPs (about 16%) in comparison with other high-income countries [15, 16]. Additional attempts to strengthen PC have focused on payment systems, including the introduction of pay for performance (P4P). Similar to the introduction of choice and privatisation, the effects on the quality of care following the introduction of P4P have been modest [17–19], in line with evidence from studies of P4P in other settings [20–22]. Moreover, studies in other countries have shown that financial incentives linked to quality measures can cause unintended effects, such as reduced doctor-patient continuity and reduced attention to activities and patients' concerns not linked to financial incentives [23, 24]. Additional studies report criticism from employees due to ethical conflicts and the perceived change of the nature of the consultations [25–27].

Criticism from GPs and other employees when it comes to “new public management” in general and extensive use of financial incentives in particular has contributed to an emerging shift in the governance of Swedish PC services [28, 29]. So-called trust-based management (“tillitsbaserad styrning”) with a greater emphasis on professional autonomy and less reliance on financial incentives has been suggested as an alternative to financial incentives [28]. In trust-based management, improved dialogue between payers and providers is emphasised, as is the responsibility of health centres (HC) for continuous quality improvement (QI) [29].

A common method to support and develop motivation for QI across care providers is audit and feedback (A&F). Brehaut defines A&F as “a summary of clinical performance (audit) over a specific period of time, and the provision of that summary (feedback) to individual practitioners, teams, or healthcare organisations” [30]. A prerequisite for A&F is access to good data. Quality improvement, including use of the Plan-Do-Study-Act (PDSA) method, has a long history in Swedish primary care [31, 32] although easily accessible data about clinical performance in PC has been limited in comparison with specialised care. “Primary Care Quality” is a new national A&F initiative in Swedish PC, developed by PC professionals, that may enable a stronger focus on QI concerning clinical quality through improved access to data obtained directly from the electronic medical records (EMRs) at the HCs. “Primary Care Quality” consists of 150 + quality indicators that reflect a wide range of PC activities, including acute and chronic conditions, rehabilitation, support of patients’ lifestyle habits, multimorbidity and continuity. The purpose is to support QI in the HCs through analysis, reflection and learning based on follow-up and comparison of data.

Currently, the “Primary Care Quality” system has been introduced at about 80% of Swedish HCs, but far from all of them have started to use the system [33, 34]. Although “Primary Care Quality” provides HCs with information about current quality several barriers exist that may prevent data from being used for QI.

The information given by A&F must also influence the intentions (willingness) to change the way of working among professionals and HC managers. Providing A&F is one way to influence this information – intent gap, but the effects vary [35]. A previous Cochrane Review found that the impact depends on the source of A&F (a higher impact with a respected colleague or supervisor), the frequency (a higher impact with repetitive A&F using new data), the improvement strategies (a higher impact with goal setting and action planning), the baseline performance (a higher impact if performance is low), and aim (a higher impact if the aim is to decrease undesirable behaviour rather than increase desirable behaviour) [36]. Additionally, a multi-modal form of feedback (combination of verbal and written/visualised form) has a positive impact on the effectiveness of A&F [36, 37]. Moreover, Colquhoun et.al. identified the importance of factors such as to whom the feedback is delivered (individual care provider, group), the type of information (process or outcome, individual or group level), and why the feedback takes place (the theoretical rationale or purpose of the feedback) [38, 39].

Even with carefully designed A&F practices, barriers related to contextual conditions may prevent QI efforts. Thus, knowledge about contextual conditions and barriers at HC level is fundamental for A&F to be able to support QI based on clinical performance data. Without such knowledge, improvements in A&F may facilitate good intentions but fail to accomplish real change.

The purpose of this study was to explore how professionals and HC managers in Swedish PC experience existing forms of A&F and present conditions and barriers for QI. We also explore views concerning the future use of clinical performance data for QI.

## Method

### Study design

The study design has an explorative and qualitative approach using focus group interviews with HC managers, physicians, and other health professionals at participating health centres for data collection. The focus group method was selected because the dynamics and interaction between participants facilitate exploration of experiences, reasoning and opinions [40, 41]. Qualitative content analysis was used to analyse transcripts of the interviews [42, 43].

### Setting

HCs are contracted by one of the 21 semi-autonomous regions responsible for the financing and organisation of health care in Sweden. Citizens can register with any contracted HC in their region, with minimal possibilities for health centres to refuse registration [44]. About 60 percent of Swedish HCs are public but the public/private mix varies between regions and depends on population density. Payment systems for HCs also vary across regions but are mainly based on risk-adjusted capitation (based on burden of disease, e.g. using diagnoses, in combination with socioeconomic factors) [45] with small fee-for-service and P4P components. The funding covers direct expenses for employees’ salaries and facilities but also indirect expenses for prescribed medications, laboratory tests and diagnostic procedures (e.g. x-rays, CT scans and ultrasound). Financial charges apply if registered patients seek PC at other PC facilities. Patient fees are moderate, about 20 € for an adult GP visit in 2020 and free for children. HCs usually register 5 000 to 15 000 persons and employ GPs who work in collaboration with practice nurses, psychologists, physiotherapists, occupational therapists and administrators. In public HCs, managers are employed and usually also a GP or a nurse. If the HC manager is not a GP, a medical officer (GP) is responsible for different aspects of care quality.

### Participants

A written invitation with information about the study was sent out to 20 HCs in two medium sized regions where 29% of HCs were private. We invited an even mix of HCs with different types of ownership and size, located in both rural and urban areas, and in geographic areas of varying socioeconomic status. We asked for participants who worked with patients and with different occupational backgrounds and experience, including HC managers. One private and six public HCs agreed to participate. Four of the participating HCs had more than 10 000 persons registered. One of the HCs was located in a larger community (> 100 000 inhabitants) one in a small community (< 10 000 inhabitants) and the rest were located in midsized communities (10 000–100 000 inhabitants). This meant that the final sample reflected the variation in the regions well in terms of ownership and size of HCs, while the selection was somewhat skewed in terms of geography, with too few HCs from larger municipalities. The socioeconomic index for the participating HCs was slightly lower than for the HCs in the regions in general. The participating HCs had in total about 14% of the population in the two regions registered. At two of the HCs, the focus groups with the HC management were held separately. At the other five HCs the managers were included in mixed focus groups. The focus group sessions were held in the HCs’ facility. Most groups had five to seven participants but one had two and one 18. In total, 17 men and 35 women participated, including all types of staff employed at the HCs: GPs, GP trainees, district nurses, assistant nurses, physiotherapists, occupational therapists, psychologists, and administrators. The managers were doctors or nurses. The participants’ working experience ranged from 0 to 40 years.

### Data collection and analysis

Two of the authors (EA and SD) jointly conducted the focus groups, one acting as moderator, one as assistant. Both had previous experience of leading focus groups. Before the discussion started, the moderator informed the participants about the study, that participation was voluntary and that information would be treated anonymously. The participants gave both verbal and written consent to participate.

We used a semi-structured interview guide with open questions, developed for the study. The topics were what good quality is, how it can be measured, present access to performance data, use of the data, present QI projects, problems and wishes for the future, Appendix.

During the focus groups the participants were encouraged to give examples from their own practice.

Each focus group discussion lasted about one hour, was audio recorded, and transcribed verbatim for subsequent analysis. The transcripts of the interviews were analysed using qualitative content analysis with an inductive approach [42, 43]. Initially EA and SD separately read the text several times to get a sense of the whole content. Meaning units relevant to the aim were then identified. In the next step the meaning units were condensed and labelled with a code. Subsequently codes with similar content were grouped into subcategories which were combined into categories [46–48].

After the individual coding and categorisation, two of the authors (EA and SD) compared their results, and when differences were found the results were discussed until consensus was reached. In a last step the themes were identified and discussed in the whole research group (EA, SD and AA).

## Results

We identified seven themes that described the participants’ views related to the existing systems for A&F and QI at the HCs: 1. Multiple forms of A&F with different purposes and designs, 2. Focus on revenues, costs and efficiency measures from regional managers and owners, 3. More limited attention to clinical quality, 4. Motivation from comparison and transparency, 5. More structured approaches needed for complex change, 6. Focus on avoiding quality degradation rather than quality improvement, and 7. Perceived barriers for QI. The last theme consisted of three categories: 7a. Criticism of measures – and hopes for better ones, 7b. Lack of time, and 7c. Responsible but not in control. A presentation of the content of each theme and selected informative quotes follow below.

### Multiple forms of audit and feedback with different purposes and designs

Participating HCs were exposed to multiple forms of A&F. We identified four different types of stakeholders with different stated purposes and varying designs in terms of their A&F: 1) the regional managers in their role as purchasers and funders, 2) the owners of the HCs, 3) the regional pharmaceutical committees and the Swedish strategic programme against antibiotic resistance (Strama) groups, and 4) the PC research and development (R&D) unit (in one of the two regions).

Except for general dialogue and information exchange they all had different aims and used different measures and targets, see Table 1.

**Table 1:** Characteristics of different stakeholders’ A&F

Stakeholder and role	*Regional managers (Purchasers and funding agents)*	*Owners of the HCs (public or private)*	*The regional pharmaceutical committees and Strama groups*	*The primary care R&D unit*
Aim	Control compliance to contractual obligations	Monitor revenues, expenditures and general performance	Monitor prescriptions at the HCs and stimulate QI	Facilitate learning and innovation through local improvement activities
Frequency	Annually	Often monthly	Annually	Annually
Form	Often face–to–face, sometimes by video link, sometimes sending out data reports only	Sometimes face– to–face meetings, but usually sending out data reports only	Often face–to–face meetings or video meetings, but sometimes sending out data reports only	Audit reports in combination with face–to–face meetings
Participants from HC	Usually limited to HC managers and key staff selected by the HC manager	HC managers (who distribute information to the staff)	HC managers and key staff at the HCs	HC managers and key staff at the HCs
Measures and targets	Facilities, staffing, opening hours and collaboration agreements with a focus on non–clinical measures Quality of care measures often linked to financial P4P incentives	Expenditures, utilisation of services (e.g. diagnostic procedures) and other non–clinical measures. Process measures linked to financial incentives (e.g. number of visits and waiting times)	Mainly clinical measures linked to treatment guidelines, evidence–based national targets and regional recommendations on drug use, including restrictive use of antibiotics	A combination of mainly clinical measures compiled by the R&D unit, reflecting quality of care for different patient groups. Discussion and reflection rather than goals
Descriptive quotes from focus group participants	*We have contractual follow–up by the reginal purchasers. They sent us feedback and asked us to comment on it. It can be clinical data, but it can also be about accessibility or staffing. Anything is possible. It can be for example [treatment with] anticoagulants. [And then they ask us to] tell them what we think about our results. Last time it was about patient safety. (HC B, manager)*	*Some feedback about finances, how we are doing on prescriptions, diagnostic procedures and laboratory tests. Then we also get feedback on how we are doing on medication reviews of patients taking more than 10 drugs, how many we have done, and the cost of drugs for diabetes, asthma, and COPD and what else is it …?* *(HC A, manager)*	*There has been a lot of feedback on [how we prescribe] antibiotics. Strama have been very much out visiting … to check that we follow the guidelines* *(HC C, Medical officer)*	*We have had a follow–up every year from the R&D unit and it has been valuable* *(VC E, manager)* *We do not have the energy or tools to set aside time ourselves to find out how many patients have heart failure or atrial fibrillation and [how many of them have] this treatment in that dose…* *(HC G, manager)*

### Focus on revenues, expenditures and production volume from owners and regional purchasers

Feedback using data and measures linked to revenues, volume of production and utilisation was regularly delivered from both the regional purchasers (yearly) and the owners (monthly). Examples of measures used include registration of diagnoses (which influenced risk adjusted capitation payments), number of visits, and the volume and expenditures related to diagnostic procedures and drug prescriptions. Most health professionals experienced that measures linked to revenues and volume of production was the main source of A&F. One HC manager said that the owners clarified their mission for the HCs through the financial goals.

The demands of balanced financial results across HCs from owners were perceived as a driving force to use the delivered data and to contain expenditures. At one HC the manager had tried to calculate and compare each doctor's production costs. However, using data linked to revenues and costs was rarely described as a positive driver by health professionals.

Several participants, including both HC managers and staff, felt that the payment system in its current form forced them to perform tasks that did not add value for patients, in order to obtain sufficient funding for activities that *did* create patient benefits in the following step. Examples of such tasks connected to funding were home visits to patients with more limited needs, carrying out QI projects not considered as necessary, and several tasks described as administrative.

*We can do some things just to get funding to be able to use the money for our patients in the next step, for example registration of diagnoses for the ACG system. We do put resources into it to be able to get better quality for our patients, but it is a detour, so to speak. It can be quite frustrating sometimes! (HC A, manager)*

Several HC managers said that it was an informal duty to act as a "filter" between employees and feedback reports from regional purchasers and owners. They studied the data and assessed the results and then brought up only the most important issues with employees.

### More limited attention to clinical quality

Participants defined clinical quality as measures reflecting the actual care provided to patients. This quality was related to correct use of procedures such as diagnostics, treatment and rehabilitation for patient groups with different diseases or needs. Quality in this sense was monitored only to a limited extent by regional purchasers and owners, with the exception of a few measures linked to P4P, e.g. proper use of antibiotics.

In contrast, the R&D unit, the pharmaceutical committee and Strama tried to focus their feedback on clinical quality when they visited the HCs. The visualisation of clinical results, and comparison with others, together with a dialogue around data and measures was perceived as valuable and inspirational for QI. The participants particularly appreciated visits by senior colleagues with experience from work at health centres.

*Then he showed how the different colleagues worked and it led to a very creative discussion between us colleagues. (HC F, Employee)*

However, most of the data on clinical quality had to be retrieved from the EMRs manually at the HCs. For example, at one HC, data on heart failure was previously studied by a GP trainee, and the HC kept following this from time to time but not regularly.

### Motivation from comparison and transparency

Working with measures and data was described as a meaningful and interesting task, particularly by medical officers and HC managers at some HCs.

A common method of using data for QI was to simply visualise it and compare with other HCs, especially for clinical data and data on utilisation of laboratory tests and diagnostic procedures.

*As soon as you raise a problem area, it suddenly just gets better. You do not have to make any changes, just raise the subject and something happens in the group and then the data becomes better. We have seen it so many times. (HC E, manager)*

For prescriptions of antibiotics and other drugs, the same method was used but with data and comparisons for each individual doctor. First, the results were discussed in a group. Then, each GP was handed a list of their patients with the expectation that each GP should “act and change their habits” accordingly.

*We focus on one topic, discuss, think about how we can improve and then we receive some statistics that are individual. And then we handle the statistics on an individual level. That's how we work. (HC D, Employee)*

The participants described both positive and negative feelings concerning sharing data that identified them as individual team members, and about comparing themselves with each other openly. Doctors were used to discussing differences of opinion, e.g. concerning the use of laboratory tests or drug prescriptions. They explained that they did not criticise or judge each other. Instead, there was an understanding of "necessary variation" linked to individual patient needs.

Nurses also monitored quality on e.g. diabetes care through the National Diabetes register (NDR) [49]. However, nurses at some HCs were not comfortable comparing their individual performance openly with others’.

For more complex problems that required collaboration and teamwork the individual feedback could be one of the steps in the process of QI. For example, one HC tried to address the issue of frequent visitors. In this case GPs also received a list of their own frequent visitors to improve the quality of care for their patients. But before this step was implemented it was decided that one GP would interview selected patients to better understand the causes of frequent visits. This led to new knowledge that was shared with other GPs before they tried to improve care for their own patients.

### More structured approaches needed for complex change

For major improvement projects, requiring organisational changes, the participants described a more structured approach using more or less formal Plan-Do-Study-Act (PDSA) cycles.

*For example, when we want the asthma nurse (instead of the doctor) to take all asthma patients who have no other disease. Then maybe more time is needed for her and we have to plan. It requires more structure and organisational changes. (HC H, medical officer)*

Some participants described how working according to the traditional PDSA method included forming an improvement team, setting goals, analysing problems, extracting data, analysing data, and considering possible changes. Subsequently the teams tested changes, measured again, evaluated outcomes and finally they proceeded to “run another lap”. Frequently, HCs used simplified and accelerated versions of the PDSA method. At one HC the manager said that they did not have time to use the method as intended. In order to speed up of the process, time spent on analysis and administration was reduced.

*We sit down, but sometimes maybe only for 15 minutes and then somebody gets up, grabs a pen and goes to the flip board. Let’s ask 10 patients who are leaving… We will meet again in two weeks. Then we’re done with analysing and move on. (HC G, manager)*

Some focus group participants described how coaching and project management from an external party could facilitate the QI process. However, coaching needed to take the conditions and priorities of the HC into account and focus on achieving results rather than on compliance with a particular method.

### Focus on avoiding quality degradation rather than improvement

Many QI projects across participating HCs were described as trying to “adapt to reality” and as avoiding quality degradation rather than accomplishing quality improvements. An important constraining contextual factor repeatedly referred to was lack of resources, not least a shortage of GPs and other health professionals. The staff searched for opportunities to increase efficiency by balancing the care provided across patient groups with different health care needs. For patients with chronic diseases, the improvement projects were about prioritising those with greater needs, e.g. by improving continuity of care and extending teamwork around patients. Improvements to drug treatment in chronic care were common, partly because they were connected to P4P funding. For patients with non-severe acute diseases, like minor infections and smaller injuries, participants described QI projects such as improving telephone accessibility and implementation of new digitally supported triage systems in order to ration the utilisation of GPs.

At all HCs, the staff tried to increase capacity by involving all professions in direct patient work through task-shifting. This included making all professions a possible "first-line contact", e.g. by enabling patients to go directly to physiotherapists, psychologists or psychosocial teams without a GP referral. HCs also arranged for nurses to take over the diagnostics and treatment of some patients from the GPs, e.g. patients with minor infections. Similarly, all professions were involved in the treatment and follow-up of patients with chronic disease.

Efforts to follow up possible negative effects of implemented projects were also described by participants. One HC planned to monitor effects by using “Primary Care Quality” to ensure that clinical quality did not deteriorate after an improvement project including task-shifting was carried out. An example from another HC was to extract data to track possible adverse effects for other patient groups than those being targeted in a particular improvement project. The HC had been criticised by regional purchasers for making patients with acute minor problems wait, and they thought that “proving” that 1–2 weeks of waiting for these patients did not cause any medical harm might lead to acceptance of their policy on prioritisation.

### Barriers to QI

#### 7a. Criticism of measures – and hopes for better ones

The participants were critical about the dominance of A&F based on measures reflecting revenues, expenditures and the volume of care.

Also measures of accessibility such as the number of patients who got an appointment within three or five days, a measure the regional purchasers found important, were often described as “pointless”.

*The law says that we should prioritise those with the greatest need for care… I think these types of measure and target create so much frustration in primary care, when much more important measures exist (HC G, manager)*

The participants’ main wish was for more measures reflecting clinical quality, including patient outcomes. They also wanted additional measures representing the patients’ perspective on care, and measures that reflected quality in PC from a broader perspective.

Many participants also said that “real quality” could not be measured. Hence, they had to accept “proxies” that could give a hint about quality even if this was not the whole truth.

*Some things you can put into numbers. These things are assessed more using soft data. It may not be as easy to measure. (HC E, manager)*

#### 7b. Responsible but not in control

Funding of HCs in the form of capitation payment had to cover both GP and other staff salaries as well as indirect expenditures related to prescribed medication, laboratory analyses and diagnostic procedures referred to by GPs. QI could increase expenditures for the HC. For example, one HC referred to QI work on optimising drug treatment for patients with diabetes, which resulted in better blood pressure and blood sugar levels for their patients but also use of more expensive medicines. This in turn led to higher expenditures for HCs, who had a financial responsibility for prescribed drugs, and increased difficulties when trying to balance revenues and costs.

A main problem, brought up by many participants, was the large and growing pressure for more PC services from different external parties. This included trying to adapt to the demands and needs of all current patients while at the same time constantly being assigned new tasks, like requests for prompt follow-ups of patients discharged from the hospital, or taking over responsibility for patient groups that used to be taken care of by hospital-based specialists, usually without getting any extra resources. The combination of resource and staff shortages, growing pressures to increase both the scale and scope of activities, and existing regulations prohibiting HCs from refusing registration of new patients was described as a “mission impossible”.

*We take care of 13,000 patients. Whether we have staff or not we are supposed to fulfil the assignment. It is like saying that a ship needs 35 crew members with different skills, and then there is only half of the crew, but the ship should sail anyway, the crew should do all these jobs. Somewhere this equation doesn’t add up. (HC F, Employee)*

Several participants experienced a conflict between what they thought was good PC quality on the one hand, and measures and targets used by regional purchasers and owners related to access to care for minor health care needs on the other hand. A solution considered necessary in order to create improved PC quality was acceptance of priority setting and rationing, i.e. to remove tasks and responsibilities in order to create a balance between what could be expected from PC activities and available resources. Such a change was not possible for a single HC, but required contractual changes with implications for all HCs in the region. Similarly, for other complex problems, QI focusing on individual HCs was deemed insufficient. To initiate more significant changes and improvements, other health care providers needed to be involved as well. Many QI projects needed to be initiated and governed from the meso level, and included both hospital and primary care. One example was patients with mental health problems where a single HC found it difficult to establish a QI project with the psychiatric clinic and involving other HCs.

#### 7c. Lack of time

Most of the participants described a desire to improve daily work at the HC, but it was difficult to find time for QI projects since their days were filled with patient appointments. The lack of time also made it difficult to obtain and assess data. The HC administrators, who had access to the relevant data, were often occupied with other tasks, such as meeting regional requirements that the doctor's dictated notes should be typed within 48 h, or the HC could lose some of its funding.

Participants at HCs also pointed out that it was difficult to follow and improve quality in several fields simultaneously, and they were not able to work as systematically as they wanted. Many emphasised that they did not just need time for the QI projects themselves, but also time to think and reflect on what they needed to improve.

*We must have enough health professionals. Because we are too few and work is too stressful, we can’t think of quality and QI projects. I think that doctors should have some time for reflection as well because it is also a part of our job to think about what we do and how we are doing it. When we are this few, maybe we need it even more. (HC C, medical officer)*

The current situation was perceived to cause frustration but was also described as a motivation to initiate certain types of QI. For example, projects could try to find ways to improve overbooked schedules for GPs or to reduce expectations on GPs to prescribe medication or order tests without direct contact with the patients.

## Discussion

Access to data for QI within PC is a general problem, although a majority of Swedish GPs report that they regularly receive at least some feedback on activities and results [50]. Our study identified four parallel forms of A&F in Swedish PC. Most of the data and feedback messages from regional purchasers and owners to HC managers and professionals concerned non-clinical measures related to revenues, expenditures, utilisation of resources and the volume of production. Measures and targets used by both regional purchasers and managers were often linked to financial incentives and contributed to fulfilling HCs’ contractual obligations. In this respect, the HC managers acted as a filter and prioritised between different demands and problems raised by regional purchasers and owners. Still, the health professionals who participated in our focus groups did not perceive that these A&F practices contributed to improved quality in general. The participants described how they needed to adapt to financial incentives to be able to raise revenues in order maintain staff levels so they could do the things they themselves found valuable for patient benefits. They also needed to keep up with the rest of the HCs in the region, e.g. when it came to registration of diagnoses and other activities related to funding, or else they would not be able to maintain their current staff level. This meant that the HCs’ financial position was an important condition for QI. HCs that did not have a good financial position felt forced to focus on improvements that had an impact on expenditures and/or revenues. On several occasions, participants referred to administrative changes that had a positive impact on HC revenues but without providing benefits for patients. The privatization and choice reforms introduced some 10 years ago may have contributed more to increased competition for revenues and resources, which explains the limited impact on PC quality [14].

In contrast to A&F from regional purchasers and owners, A&F based on clinical data by the regional Pharmaceutical committee/Strama group and the regional R&D unit was often described as meaningful and motivational by participating professionals. This is in line with previous studies suggesting that A&F is more likely to be accepted if professionals trust data, agree with benchmarks and/or consider clinical topics being audited important [35, 51, 52]. The staff at the HCs asked for more A&F focusing on measures related to their clinical quality in comparison with other HCs and with evidence-based targets. The participants wanted easily accessible clinical data highlighting the content and results of care, preferably in the form of recurring reports and taking into account patient experiences and outcomes. The clear dominance of non-clinical data in A&F from regional purchasers and owners was described as an important barrier to such QI work based on clinical data. A&F in Swedish PC started as a voluntary intra-professional activity, where GPs assessed their own work with the purpose of sharing experiences and initiating improvement work [32]. More recently, and as components of new public government (NPM) reforms, A&F by regional purchasers and owners has been implemented. This form of A&F focuses on external accountability, regulations and sanctions [53, 54] and is exercised by individuals that are not personally involved in the work at the HC. The motivation to respond to feedback messages is based on external incentives rather than on professionals´ motivation. Our study suggests that a balance is required between different forms of A&F. An unbalanced practice of A&F, focusing on revenues, expenditures and non-clinical measures, is likely to suppress A&F based on clinical data and may reduce professionals’ interest in A&F activities in general. To find a new balance between internal and external A&F, where the external does not dominate the internal, can be described as an essential condition for more QI work and use of clinical data.

Facilitating access to relevant clinical data for PC is described as a necessary first step in the process of supporting QI [55]. The evidence-based quality indicators in Primary Care Quality are easily integrated into the daily work at HCs and can be an important contribution towards this end in Swedish PC [55]. Improved access to clinical data also needs to be translated into information, e.g. using graphical presentations and comparisons with evidence-based targets, and benchmarking against peers to trigger motivation and engagement [55, 56]. Our participants, and in particular GPs, confirmed that comparisons of data between HCs stimulated QI. GPs frequently discussed their individual results openly with each other and felt that they could do so non-judgmentally. The nurses, on the other hand, who did not have much experience, were less comfortable with comparison of results. According to previous studies, the motivation to improve is likely to increase if A&F is delivered by a trustful source and someone professionals look up to [57]. A dialogue, with self-reflection on results, is suggested to make it easier to include some of the complexity, and the non-measurable values in PC, in the evaluation of quality [58]. This method of “collegial dialogues” had a long history in one of the studied regions and was perceived as valuable by the participants in our study. The Swedish Strama groups have a similar model of colleagues visiting HCs that has proven successful [59]. Additional conditions to facilitate QI work concern ownership. Improvement projects that health professionals experience as “their own” have a greater chance of success, as autonomy along with having a professional ideal of pursuing mastery and a clear and meaningful social purpose, creates motivation in itself [57, 58].

The HC managers in our study were usually less active in QI work based on clinical data. To a high degree, this work was left to individual professionals. Several changes was “simple” as each GP could implement changes individually, e.g. related to more restricted prescriptions of antibiotics by GPs. More complex changes require a team effort and a more active role for HC managers. Such changes are also more likely to need methodological support, e.g. using Plan-Do-Study-Act (PDSA) cycles [60]. However, studies show that the PDSA model is rarely used as intended in practice [61]. In fact, a recent study from the UK reported that only 20% of GPs and 33% of practice managers knew about the PDSA model [62]. Financial incentives for using PDSA in QI in Swedish PC have been tested [63] but the impact on the care provided is yet to be studied. Although they accepted the need for external support, the participants in our study stressed the need to adapt the methods of QI to their own needs and capabilities, and to focus on results rather than strict compliance with methods. This is in line with other studies, pointing out the risk of having too much focus on tools for QI when it is”the improvement habit” along with knowledge and skills in providing care that truly matters [64]. Even when a simplified version of the PDSA model was used across the HCs in our study, patients were involved in the QI work and contributed with their experiences and perspectives.

In practice, professionals and managers at HCs are occupied with delivering services and taking care of acute problems. They often have little time to analyse or think about data that reflects past events, or how to change current practices in an innovative way. Therefore, facilitating more complex QI work through a coach who supports the team working on improvement is common [65]. Our participants highlighted that the greatest benefit of a QI coach, besides "putting pressure" on the participants, was that they were helped with practical things such as compiling data. This indicates that the main problem was not ignorance of QI methods but a lack of time, perhaps the most important barrier for QI. Using data to implement well-thought-out QI projects that actually lead to improved outcomes for patients takes time [66–68]. In particular, time constraints may be an important barrier for complex changes requiring collective efforts at the team level. Such changes requires an adaptive approach, accepting unpredictability, and self-organisation including time for reflection [53, 54]. The participants in our study expressed a need not only for time to implement QI projects, but also time to reflect on what needed improvement. The authors of a Health Foundation report state that “Improvement teams that try to cram the planning of a complex intervention involving multiple processes and people into a few brief impromptu meetings held between clinical commitments will struggle to make an impact.” [65]. Furthermore, previous experiences of lack of resources (time and staff) during attempts to perform QI projects can also cause change fatigue and decreased motivation [69, 70].

The necessary conditions for QI, and in particular complex change, are in sharp contrast to existing conditions in PC. According to a survey by the Health Foundation, 95% of responding GPs in both Sweden and the UK found their work stressful, and more than half of them found it very or extremely stressful [71]. In the UK, 80% of responding GPs found it a major challenge to find time to plan and design improvement projects. In Sweden, where about half of all HCs have vacancies, there is no reason to believe that the situation is different [72]. These conditions also explain our finding that many QI projects across the studied HCs were about avoiding quality deterioration rather than improvement. The situation is also reflected in the participants' desire to prioritise patients with greater health care needs, and their request to receive support for giving patients with minor health care needs a lower priority. In fact, focusing on QI and cost reduction, without considering work conditions for staff, may have a negative impact on results [70].

The participants in our study often described to themselves as having responsibility but not being fully in control. This points to the importance of working with QI on a broader system level that includes other health care providers, especially if a transformational change in support of a PC-based health system is expected. A previous IHI paper highlights systems thinking and the need to collaborate across provider boundaries to achieve such ends [73]. To make change at the system level possible, it is essential that leaders at the macro level have a good knowledge of the system, but it is also essential to strengthen leader’s capabilities and motivation to initiate and implement change [74].

### Limitations

Our study has several limitations. A first important limitation is selection bias since participation in the focus groups was voluntary. It is possible that participating HCs had a greater interest in QI compared to HCs that did not participate. Non-participating HCs may also have more time constraints. If anything, this suggests that the conditions and barriers reported in the study regarding lack of time may be underestimated. We invited HCs of different locations, sizes and ownership, but only one private HC participated. It is possible that having more private HCs with different ownerships could have provided additional and valuable information. Information bias is also possible since the number of participants was too high or low to create the best conditions for a dynamic and interactive dialogue between participants in two of the groups. However, the participants knew each other, which made the atmosphere relaxed and gave everybody an opportunity to talk, even in the big group. A general limitation is that our results are qualitative and originate from a Swedish context. The results are not directly transferable to other settings, although team-based care is common in many other countries. PC systems in other countries also face similar problems and challenges as in Sweden, including shortages of GPs and other PC professionals.

## Conclusion

Our study describes experiences with existing forms of A&F as well as views on conditions and barriers for QI among managers and professionals at participating Swedish HCs. We found four different types of A&F at the HCs, a dominance of non-clinical measures and use of targets linked to financial incentives focusing on external accountability from regional purchasers and owners of HCs. The participants in our focus groups often expressed dissatisfaction with the present situation. The dominance of non-clinical measures and a focus on external accountability were perceived as barriers to QI by health professionals. Other important barriers were lack of time, autonomy and ownership of QI work. QI was sometimes described as avoiding quality degradation rather than improving quality. This was explained by resource constraints and pressing demands on services.

In spite of barriers and poor conditions, health professionals at all HCs were engaged in at least some QI work. They wanted more clinical data and data capturing patients’ views and experiences. They also wanted more appropriate and supportive regulation, including adequate resources and time to engage in QI work. Professionals and HC managers also called for more QI work at the system (macro) level, in support of a transformation towards a primary care-based system.

## Data Availability

The datasets analysed during the current study are available from the corresponding author on reasonable request.

## Interview guide

**What is good quality in your work?**

- Can it be measured in numbers?
- Does it need to be measured?

**What do you know about the quality of your work (at your VC)?**

- Can you retrieve data yourself?
- Do you receive data reports (feedback on results) in any form? About what?
- What would you like to know about your work? Is there data you lack?
  **What would you like to know about your work?**
- What type of data reports (feedback on results) do you receive?
- Is there data you lack?
- Advantages and disadvantages of data reports?

**How do you use information about the quality of your work?**

- Do you use them in any way? Why / why not? (Previous experience? Examples?)
- Who? Who uses the data? Do all staff take part? (Discuss in meetings, put on a noticeboard?)
- How do you use them? Please, give examples based on your own experience.

**How do you work with quality improvement?**

- How do you usually choose an area?
- Where does the initiative come from?
- Are the data reports used for any type of quality improvement?
- Do you work on quality improvement in any other way?
- Who participates?
- Is there anyone who is extra knowledgeable / who takes the lead?

**How does the work with quality improvement work?**

- What makes you do / not do improvement work?
- What enables / complicates the work?
- What support have you received / needed?
- What happens in the long run with quality improvement work? Is it sustainable? How do you follow up on projects over time?

**How can QI, A&F and the use of performance data be improved?**

- What is required for data to be useful in quality improvement?
- What would you need help with? (time, knowledge, commitment, coaching, other)

For all questions: Can you tell us more? Examples? Describe how: What happens next? How do you proceed?

## Abbreviations

A&F: Audit and feedback
EMR: Electronic medical records
GP: General practitioner
HC: Health (care) centre
NDR: National Diabetes register
P4P: Pay for performance
PDSA: Plan-Do-Study-Act
PC: Primary care
QI: Quality improvement
R&D: Research and development
Strama: The Swedish strategic programme against antibiotic resistance
